# Chlorella Induces Stomatal Closure via NADPH Oxidase-Dependent ROS Production and Its Effects on Instantaneous Water Use Efficiency in *Vicia faba*

**DOI:** 10.1371/journal.pone.0093290

**Published:** 2014-03-31

**Authors:** Yan Li, Shan-Shan Xu, Jing Gao, Sha Pan, Gen-Xuan Wang

**Affiliations:** College of Life Sciences, Zhejiang University, Hangzhou, China; Key Laboratory of Horticultural Plant Biology (MOE), China

## Abstract

Reactive oxygen species (ROS) have been established to participate in stomatal closure induced by live microbes and microbe-associated molecular patterns (MAMPs). Chlorella as a beneficial microorganism can be expected to trigger stomatal closure via ROS production. Here, we reported that Chlorella induced stomatal closure in a dose-and time-dependent manner in epidermal peels of *Vicia faba*. Using pharmacological methods in this work, we found that the Chlorella-induced stomatal closure was almost completely abolished by a hydrogen peroxide (H_2_O_2_) scavenger, catalase (CAT), significantly suppressed by an NADPH oxidase inhibitor, diphenylene iodonium chloride (DPI), and slightly affected by a peroxidase inhibitor, salicylhydroxamic acid (SHAM), suggesting that ROS production involved in Chlorella-induced stomatal closure is mainly mediated by DPI-sensitive NADPH oxidase. Additionally, Exogenous application of optimal concentrations of Chlorella suspension improved instantaneous water use efficiency (WUE_i_) in *Vicia faba* via a reduction in leaf transpiration rate (E) without a parallel reduction in net photosynthetic rate (P_n_) assessed by gas-exchange measurements. The chlorophyll fluorescence and content analysis further demonstrated that short-term use of Chlorella did not influence plant photosynthetic reactions center. These results preliminarily reveal that Chlorella can trigger stomatal closure via NADPH oxidase-dependent ROS production in epidermal strips and improve WUE_i_ in leave levels.

## Introduction

Stomata are natural microscopic pores located in the epidermis of the aerial parts of plants that play a pivotal role in regulating influx of CO_2_ for photosynthesis and water loss through transpiration [1]. However, transpiration and photosynthesis do not respond to the changing stomatal aperture identically [2], [3]. As the stomatal aperture decreases above the threshold, leaf transpiration rates always diminish prior to the CO_2_ assimilation rates, favoring a higher instantaneous water use efficiency (WUE_i_) [3]–[5]. It provides a basis for the suggestions that partial closure of the stomata might conduce to the improvement of WUE_i_ in plants [4], [6]. It is well known that moderate stomatal closure can be triggered by numerous abiotic cues including high light intensity, high CO_2_ concentrations, low air humidity, and especially drought stress, see the review [7]. Moreover, previous studies have also shown that stomatal closure can be induced by multiple biotic cues such as pathogenic microbes and purified MAMPs [8]–[12]. However, some pathogenic microbes can reopen closed stomata via various virulence factors such as coronatine, fusicoccin and oxalic acid [9], [11], [13], [14]. Noticeably, a kind of nonpathogenic yeast *Saccharomyces cerevisiae* is observed to induce stomatal closure via ROS production mediated mainly by salicylhydroxamic acid-sensitive peroxidase in a recent study [15]. Besides yeast, whether other nonpathogenic microbes can trigger stomatal closure remains less known. Just as nonpathogenic as *Saccharomyces cerevisiae*, *Chlorella vulgaris* is a unicellular photosynthetic microorganism that can employ light energy and CO_2_, with higher photosynthetic activity than plants [16]. *Chlorella vulgaris* can synthesize many bioactive substances such as carbohydrates, proteins, function lipids, amino acids and vitamins, with positive effects both on humans and animals, as well as the improvement of seed germination capacities, root growth and nutrient uptake of cash crops [17]–[23]. However, the effects of Chlorella on stomatal movement and WUE_i_ of plants have not been studied. We therefore explore whether Chlorella can be sensed by guard cells to induce partial stomatal closure and improve WUE_i_ of plants when applied as foliar spray.

In plant cells, ROS act as key second messengers in mediating stomatal closure triggered by abiotic and biotic signals [7]–[9], [12], [24]. ROS production induced by various stimuli in guard cells is modulated by specific enzymes, including NADPH oxidase, peroxidase, xanthine oxidase, oxalate oxidase and amine oxidase. The various enzymes have different functions in distinct signaling pathways [25]–[28]. For instance, ROS production mediated by diphenylene iodonium chloride (DPI)-sensitive plasma membrane NADPH oxidase is involved in abscisic acid (ABA)-, methyl jasmonate (MeJA)-, ozone-, darkness, ethylene-, allyl isothiocyanate (AITC)-, low dose of ultraviolet B (UV-B)-, bacterial flagellum (flg22)-, bacterial elongation factor Tu (elf18)- and bacterial lipopolysaccharides (LPS)-induced stomatal closure [9], [29]–[36], while ROS production modulated by salicylhydroxamic acid (SHAM)-sensitive cell wall peroxidase is implicated in salicylic acid (SA)-, high dose of UV-B-, chitosan-, yeast elicitor (YEL)-, methylglyoxal- and yeast-triggered stomatal closure [15], [24], [37]–[41]. However, it is unknown whether Chlorella induces ROS production and what is the enzyme source of ROS production in guard cells.

Certain dicotyledon (*e.g. Vicia faba* L.) and microalgae (*e.g. Chlorella vulgaris*) provide ideal material models for exploring the effects of Chlorella on stomatal movement and WUE_i_ in *V. faba*. Using epidermal strip bioassay, H_2_O_2_ fluorescence assay, gas-exchange measurements, chlorophyll fluorescence and content analysis, we were to determine 1) whether Chlorella can be perceived by guard cell to trigger partial stomatal closure in epidermal strips of broad bean; 2) whether this action requires ROS production, and if so, which enzyme mediates ROS production; 3) whether this action improves WUE_i_ in *V. faba*.

## Materials and Methods

### Plant materials

Seeds of broad bean (*V. faba* L. cv. Da qing pi) were selected and sterilized in 70% ethanol for 30 min, then cleaned with distilled water. Sterilized Seeds were soaked in water until they were germinated, then transplanted into pots (5 cm×10 cm) containing a mixture of growing medium: vermiculite (3∶1, v/v). Plants were grown in a controlled growth chamber with a temperature of 20°C–25°C, a relative humidity of 70%, photosynthetic active radiation (PAR) of 300 µmol m^−2^ s^−1^ and a photoperiod of 14 h light/10 h dark, and watered daily. When plants were 4 weeks old, the even-aged fully expanded leaves were used as experimental material.

### Chemicals

Molecular probe 2′, 7′-dichlorofluorescin diacetate (H_2_DCF-DA, Sigma-Aldrich, St Louis, MO, USA) was dissolved in dimethyl sulfoxide to produce a stock solution, which was aliquoted. Salicylhydroxamic acid (SHAM), diphenyleneiodonium chloride (DPI), catalase (CAT, bovine liver) and ethanesulfonic acid (MES) were obtained from Sigma-Aldrich. Besides these chemicals, the remaining chemicals were purchased from Chinese companies. All the chemicals used were of the highest analytical grade.

### Culture of Chlorella cells

The Chlorella (*Chlorella vulgaris*) sample used in this study was obtained from FACHB (Wuhan, People's Republic of China). Chlorella cells were cultured in Erlenmeyer flasks (500 mL) containing 250 mL BG11 medium (PH 6.8) and shaken at 150 rpm in a rotary shaker under controlled conditions at a temperature of day 25°C/night 22°C, photosynthetic active radiation (PAR) of 50 µmol m^−2^ s^−1^ and a photoperiod of 16 h light/8 h dark. After 7 days of culture, the Chlorella cells were collected by centrifugation, and then rinsed twice with sterile water. Finally the cells were resuspended in sterile double distilled water to yield the different concentrations of Chlorella suspension (1.0×10^6^, 1.0×10^7^, 1.0×10^8^, 1.0×10^9^ and 1.0×10^10^ ind mL^−1^) as determined by optical density and serial dilutions with plate counts.

### Stomatal bioassay

Stomatal bioassay experiments were performed as described [15], [42], [43] with slight modifications. Briefly, the epidermis was first peeled carefully from the abaxial surface of the youngest, fully expanded leaves of 4-week-old plants, and cut into strips, then incubated in opening buffer (10 mM MES, 50 mM KCl, pH 6.15) for 2 h under light condition (photon flux density of 300 µmol m^−2^ s^−1^) at 22°C–25°C to promote stomatal opening. Once the stomata were fully open, the epidermal strips were transferred to opening buffer containing different concentrations of Chlorella suspension (0, 1.0×10^6^, 1.0×10^7^, 1.0×10^8^, 1.0×10^9^ and 1.0×10^10^ ind mL^−1^) for another 2 h. When inhibitors or scavenger (SHAM, DPI, or CAT) were used, they were added 30 min prior to the Chlorella treatments. Finally, stomata were digitized using a Canon PowerShot G10 camera coupled to a DSZ5000X microscope (UOP, Chongqing, China). The width and length of stomatal pore were measured on digital images with the Image-Pro plus6.0 software (Media Cybernetics, Silver Springs, MD). The stomatal aperture was calculated as pore width/length. Fifty stomata originated from three separate plants were randomly chosen for each treatment and the experiments were repeated three times [15], [36]. These data represented 150 measurements ± SE. To obtain time response course, the stomatal aperture was examined at 20 min intervals. Twenty stomata were randomly selected for three independent experiments. These data represented 60 measurements ± SE.

### Measurement of ROS in guard cells

Reactive oxygen species (ROS) in guard cells were examined by loading epidermal preparations with 2′, 7′-dichlorofluorescin diacetate (H_2_DCF-DA). After the treatment described in the above experiment, the epidermal strips were transferred to opening buffer containing 50 µM H_2_DCF-DA for 15–20 min in the dark at room temperature. The excess dye was washed out with opening buffer and fluorescence photographs of guard cells were taken with a Canon PowerShot G10 camera coupled to a DSZ5000X microscope (UOP, Chongqing, China) after 15–20 min. The acquired fluorescence images were processed using Image-Pro plus 6.0 software (Media Cybernetics, Silver Springs, MD). Average fluorescence intensities of treated cells were normalized to the control value taken as 100%. In each treatment, three epidermal strips from different plants were measured and the experiment was repeated three times. These data represented 100 measurements ± SE.

### Gas-exchange measurements and leaf stomatal bioassay

When plants were 4 weeks old, the different concentrations of Chlorella suspension (1.0×10^6^, 1.0×10^7^, 1.0×10^8^, 1.0×10^9^ and 1.0×10^10^ ind mL^−1^) and water were sprayed onto all fully expanded leaves present with a hand sprayer until the both sides of leaves were uniformly wet. After treatment for 48 h, the net photosynthetic rate (P_n_), stomatal conductance (g_s_), intercellular CO_2_ concentration (C_i_) and transpiration rate (E) of the fourteen random fully expanded leaves were measured on seven individual plants per treatment under a photosynthetic active radiation (PAR) of 1000 µmol m^−2^ s^−1^ with a portable photosynthetic open-system between 10:00 a.m. and noon (CI-340, 4845NW Camas Meadows Drive, Camas, WA, 98607, USA) [44]. Before measurements, the plants were allowed to acclimate to a sufficient light irradiance for more than 1 h. In operation, air temperature in the assimilation chamber was kept at 25°C, air flow was set to 500 mL min^−1^ and ambient CO_2_ concentration was maintained at 380 ppm. Data were recorded after 3–4 min, when the steady-state photosynthesis was achieved. WUE_i_ was calculated as P_n_/E [45].

To further test the effects of Chlorella on stomatal aperture in leaves level, the epidermal strips of expanded leaves of broad beans sprayed with different concentrations of Chlorella suspension and water for 48 h were peeled off and immediately observed under a microscope. Finally, stomata were digitized using a Canon PowerShot G10 camera coupled to a DSZ5000X microscope (UOP, Chongqing, China). The width and length of stomatal pore were measured on digital images with the Image-Pro plus6.0 software (Media Cybernetics, Silver Springs, MD). 120 stomata were randomly selected for each treatment from different epidermal strips of seven individual plants. These data represented 120 measurements ± SE.

### Chlorophyll fluorescence measurements

Maximal photochemical efficiency (F_v_/F_m_), the effective quantum yield of PSII (ΦPSII), non-photochemical quenching (NPQ), the coefficient for photochemical quenching (qP) and electron transportation rate (ETR) were measured on the same leaves used for gas exchange analysis with the MAXI version of the IMAGING-PAM M-Series chlorophyll fluorescence system (Heinz-Walz GmbH, Effeltrich, Germany). The experiment procedures and nomenclature used for fluorescence measurements were based on the descriptions in previous studies [46]. Saturating light pulses were given every 20 s [47], [48].

### Chlorophyll determination

The same leaves selected for gas-exchange and chlorophyll fluorescence analysis were used for immediate chlorophyll determination with a spectrophotometer (SP752, Shanghai, China). 0.1 g leaf tissue was first homogenized in 10 mL acetone: ethanol (1∶1, v/v) for 24 h, then the chlorophyll content of the supernatant was measured and supernatant absorption spectrum was set at 645 and 663 nm. The chlorophyll a and b content were calculated as [(12.71×A_663_)−(2.69×A_645_)]/[100×(fresh weight of leaves)] and [(22.88×A_645_)−(4.67×A_633_)]/[100×(fresh weight of leaves)], respectively. The unit was reported as mg Chl. per g FW, as described in [49], [50].

### Statistical analysis

Statistical analyses were performed using the one-way analysis of variance (ANOVA) procedure of SPSS (ANCOVA; SPSS13, SPSS Inc., Chicago IL, USA). Significant differences among treatments were based on *P* values determined using LSD test (*P*<0.05).

## Results

### Chlorella-induced stomatal closure in broad bean

**Figure 1:**
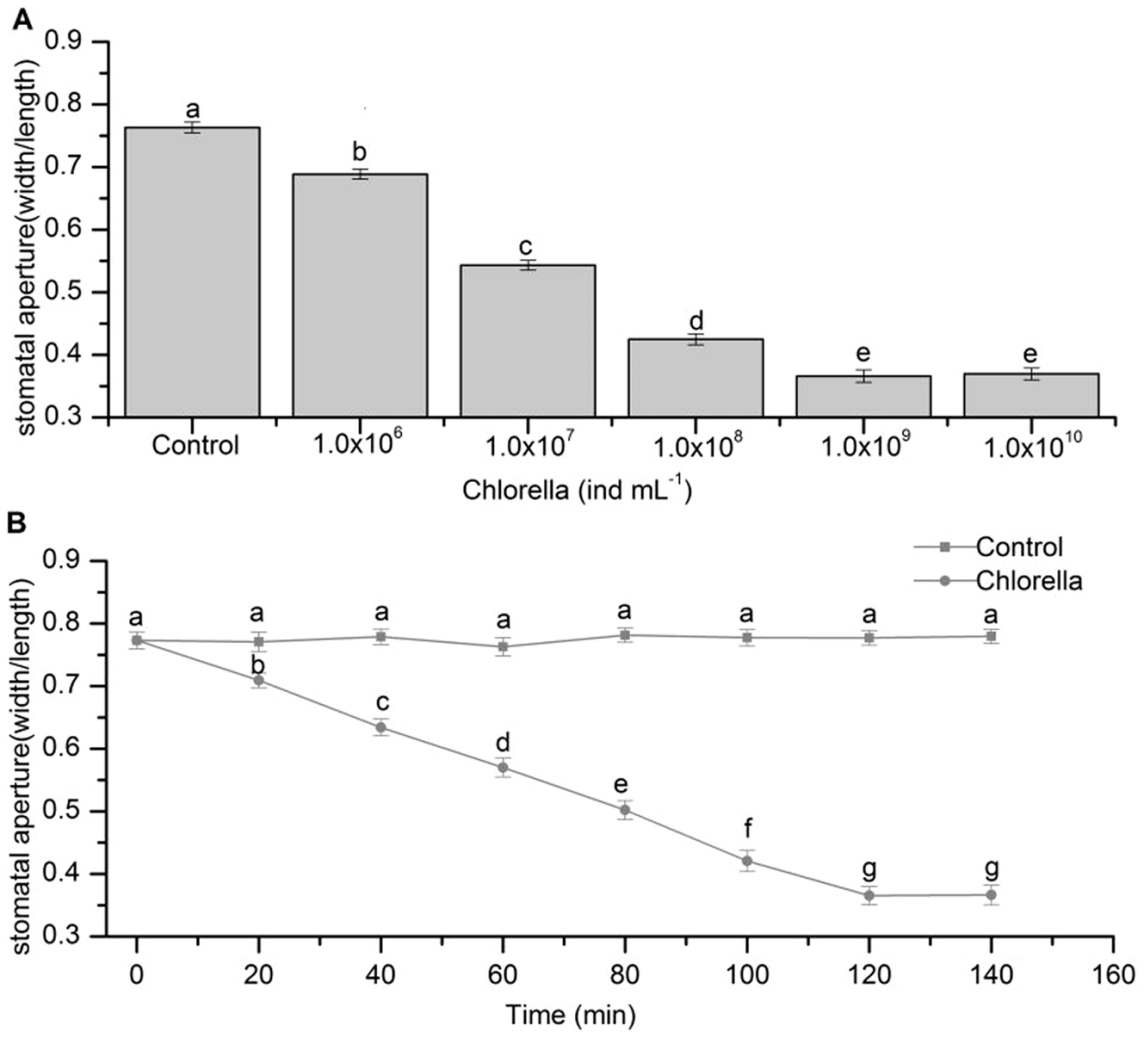
Chlorella-induced stomatal closure in *V. faba*. (A) The dosage effect of Chlorella-induced stomatal closure. Epidermal peels of broad beans preincubated for 2 h in opening buffer under light were treated with various concentrations of Chlorella suspension, and stomatal apertures were measured after 2 h. Each bar represents the mean ± SE of three biological replicates (n = 150). (B) Time response course of stomatal closure triggered by Chlorella (1.0×10^9^ ind mL^−1^) and stomatal apertures were quantified every 20 min. Data are the mean of 60 measurements ± SE from three biological repeats. Solid square: Control, solid circle: Chlorella. Means with different letters are significantly different from one another as determined by ANOVA (LSD test, *P*<0.05).

To determine whether Chlorella has any effect on stomatal movement, the abaxial epidermal peels of broad bean were separately treated with 1.0×10^6^, 1.0×10^7^, 1.0×10^8^, 1.0×10^9^ and 1.0×10^10^ ind mL^−1^ of Chlorella suspension. After 2 h, the stomatal apertures were reduced by 7.5% (*P*<0.001), 22.0% (*P*<0.001), 33.8% (*P*<0.001), 39.7% (*P*<0.001), and 39.4% (*P*<0.001), respectively, and showed a dosage effect (Figure 1A). Furthermore, as noted in Figure 1B, 1.0×10^9^ ind mL^−1^ of Chlorella suspension induced stomatal closure in a time-dependent manner, reaching the maximum effect at 2 h after treatment, under which conditions the stomatal apertures were reduced by 40.8% (*P*<0.001).

### The effects of CAT, DPI and SHAM on Chlorella-induced stomatal closure

**Figure 2:**
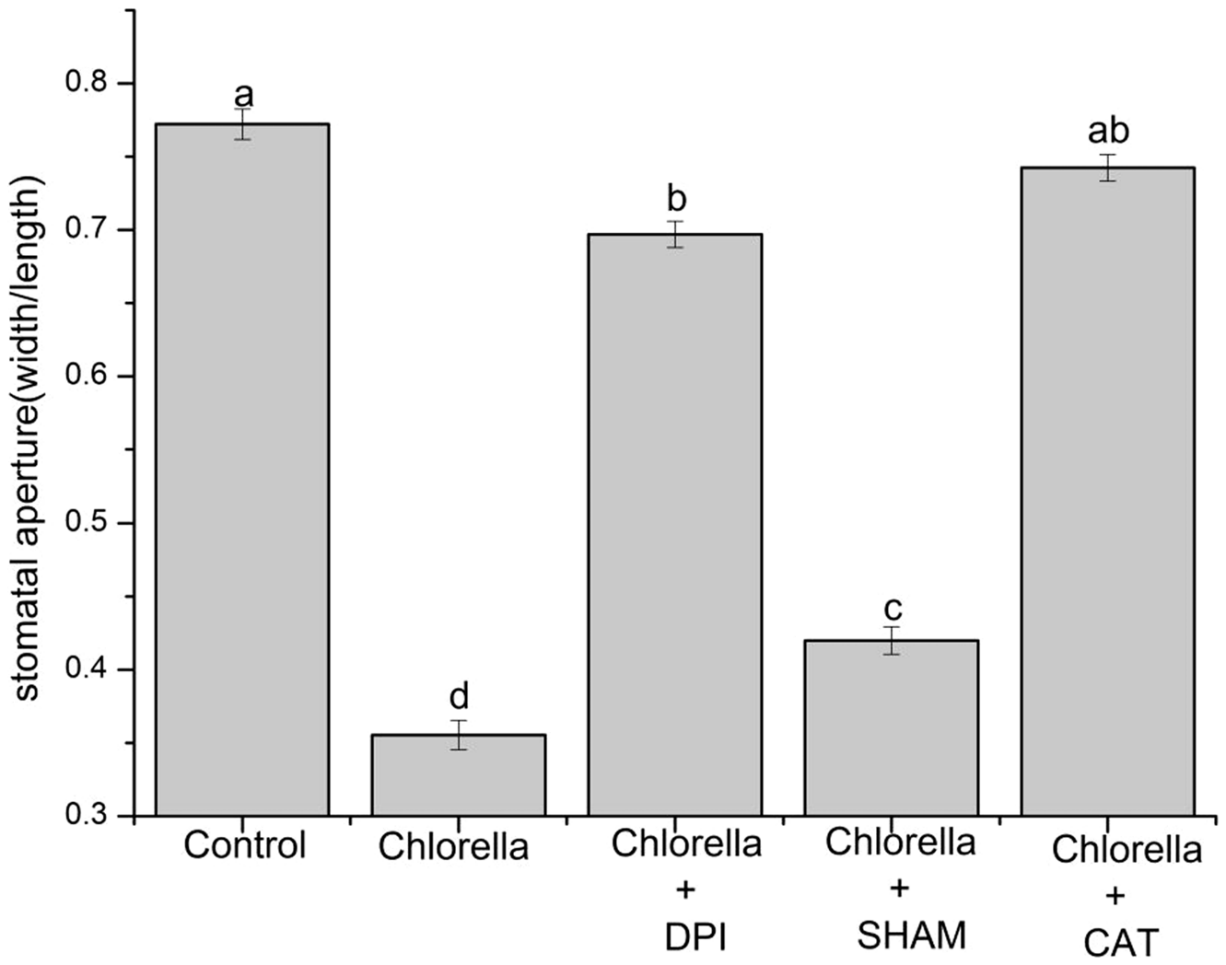
Effects of 20 µM DPI, 2 mM SHAM and 100 UmL^−1^ CAT on Chlorella-induced stomatal closure. Epidermal peels of broad beans preincubated for 2 µM DPI, 2 mM SHAM and 100 UmL^−1^ CAT for 30 min, then, floated on 1.0×10^9^ ind mL^−1^ Chlorella suspension and stomatal apertures were measured after 2 h. These data are the mean ± SE of the representative results from three biological repeats (n = 150 per bar). Different letters above the bars indicate mean values that are significantly different from one another as determined by ANOVA (LSD test, *P*<0.05).

ROS play a vital role in regulating stomatal movement [34], [40], [51]. To confirm the involvement of ROS in Chlorella-induced stomatal closure and elucidate the enzymatic sources of ROS production, the abaxial epidermal strips of broad bean were incubated with 1.0×10^9^ ind mL^−1^ of Chlorella suspension with or without CAT, DPI or SHAM, all of which remove or decrease ROS level [8], [24], [42]. We observed that Chlorella-induced stomatal closure was almost entirely restored by a H_2_O_2_ scavenger, CAT, at 100 UmL^−1^ (*P* = 0.03), strongly inhibited by an NADPH oxidase inhibitor, DPI, at 20 µM (*P*<0.001), while slightly suppressed by a peroxidase inhibitor, SHAM, at 2 mM (*P*<0.001) (Figure 2). Additionally, there was no statistical change in stomatal aperture when epidermal strips of broad bean were treated with DPI, SHAM or CAT alone (Figure S1).

### Chlorella-induced ROS production in guard cells of broad bean

**Figure 3:**
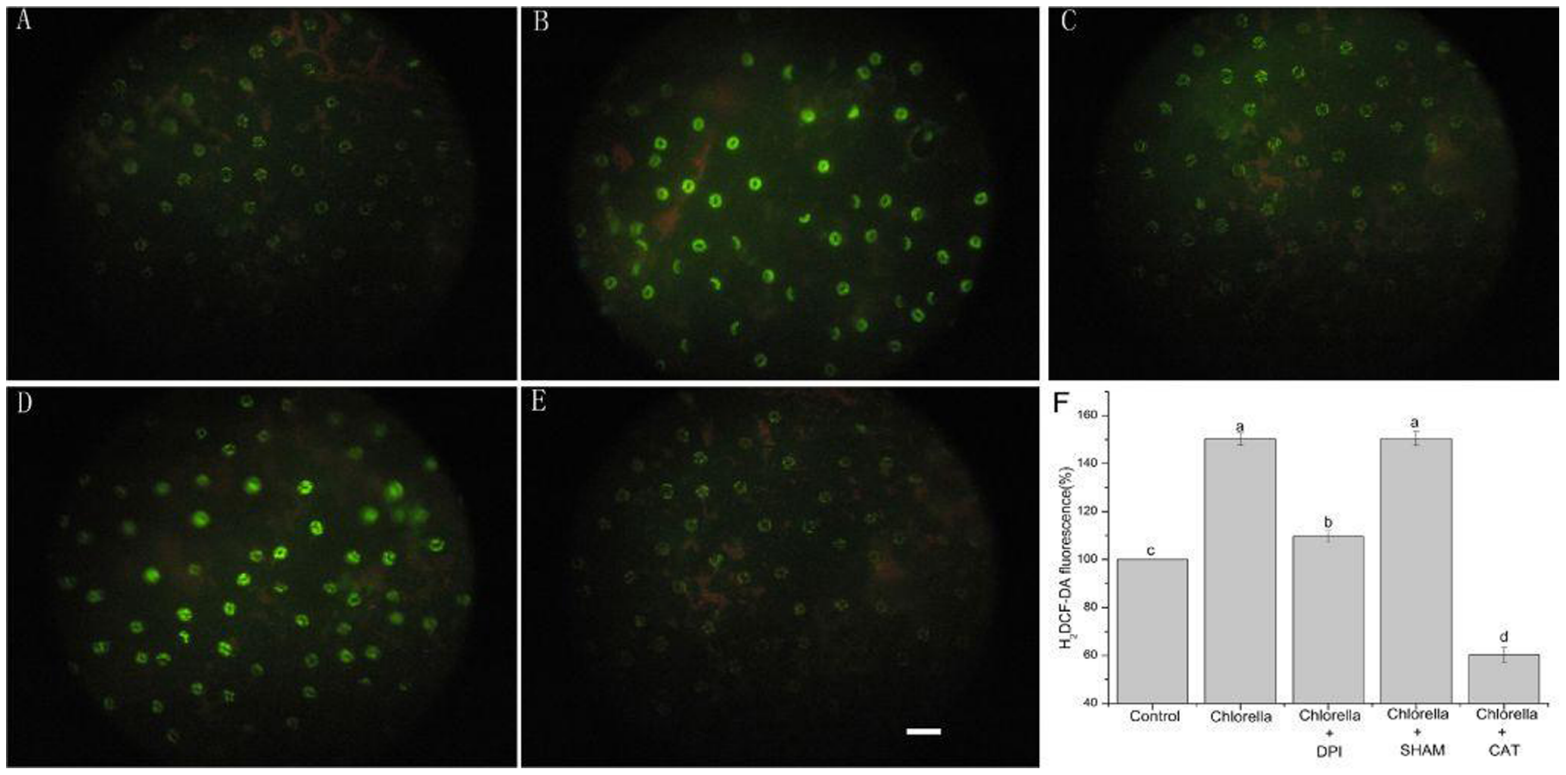
Chlorella induces ROS production in *V. faba*. Epidermal peels of broad beans without (control) (A) or with 120 min of treatment with Chlorella alone (B), with Chlorella and DPI (C), with Chlorella and SHAM (D) or with Chlorella and CAT (E) were loaded with 50 µM of H_2_DCF-DA for 15–20 min in the dark. After a brief wash with opening buffer, photographs were taken for representative pairs of guard cells from each treatment using fluorescence microscopy (A–E). The scale bar in E is 100 µm, and applies to all photographs. (F)The average fluorescence intensity of guard cells in images (A–E). The vertical scale represents percentage response relative to fluorescence value taken as 100% in control treatments. These data are the mean ± SE of the representative results from three biological repeats (100 total guard cells per bar). Different letters above the bars indicate mean values that are significantly different from one another as determined by ANOVA (LSD test, *P*<0.05).

ROS act as important second messengers in mediating stomatal closure [29], [39], [52], [53]. ROS production via NADPH oxidase has been demonstrated to participate in Chlorella-triggered stomatal closure in the stomatal bioassay experiments. Therefore, we further monitored Chlorella-induced ROS production in guard cells using 2′, 7′-dichlorofluorescin diacetate (H_2_DCF-DA). As shown in Figure 3, the ROS level in guard cells was significantly improved when 1.0×10^9^ ind mL^−1^ of Chlorella suspension was applied (*P*<0.001). Moreover, the Chlorella-induced ROS production was completely inhibited by 100 UmL^−1^ CAT (*P*<0.001), largely suppressed by 20 µM DPI (*P*<0.001) while not affected by 2 mM SHAM (*P* = 0.949) (Figure 3).

### The effects of Chlorella on gas-exchange

**Figure 4:**
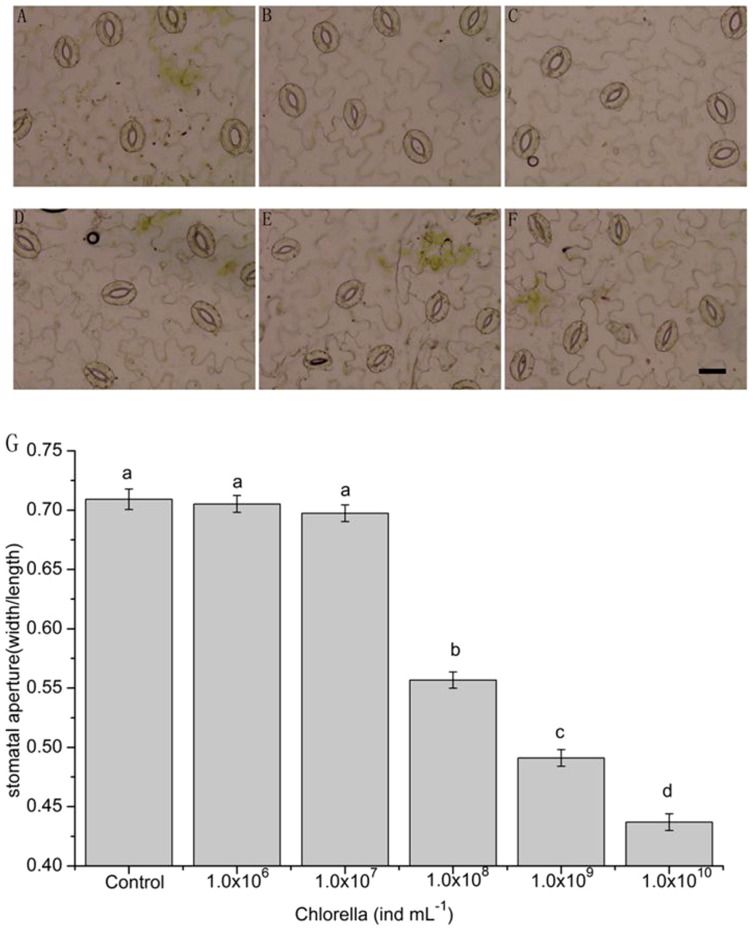
The effects of Chlorella on stomatal movement in leave level. After spraying uniformly different concentrations of Chlorella suspension (1.0×10^6^, 1.0×10^7^, 1.0×10^8^, 1.0×10^9^ and 1.0×10^10^ ind mL^−1^) and water onto the leaves of broad bean for 48 h, the epidermal strips were peeled off and immediately observed under a microscope. Photographs were taken for representative pairs of guard cells from each treatment using microscopy (A–F). The scale bar in F is 40 µm, and applies to all photographs. (G) The average stomatal aperture shown in images (A–F). These data are the mean ± SE of the representative results (n = 120 per bar). Different letters above the bars indicate mean values that are significantly different from one another as determined by ANOVA (LSD test, *P*<0.05).

**Figure 5:**
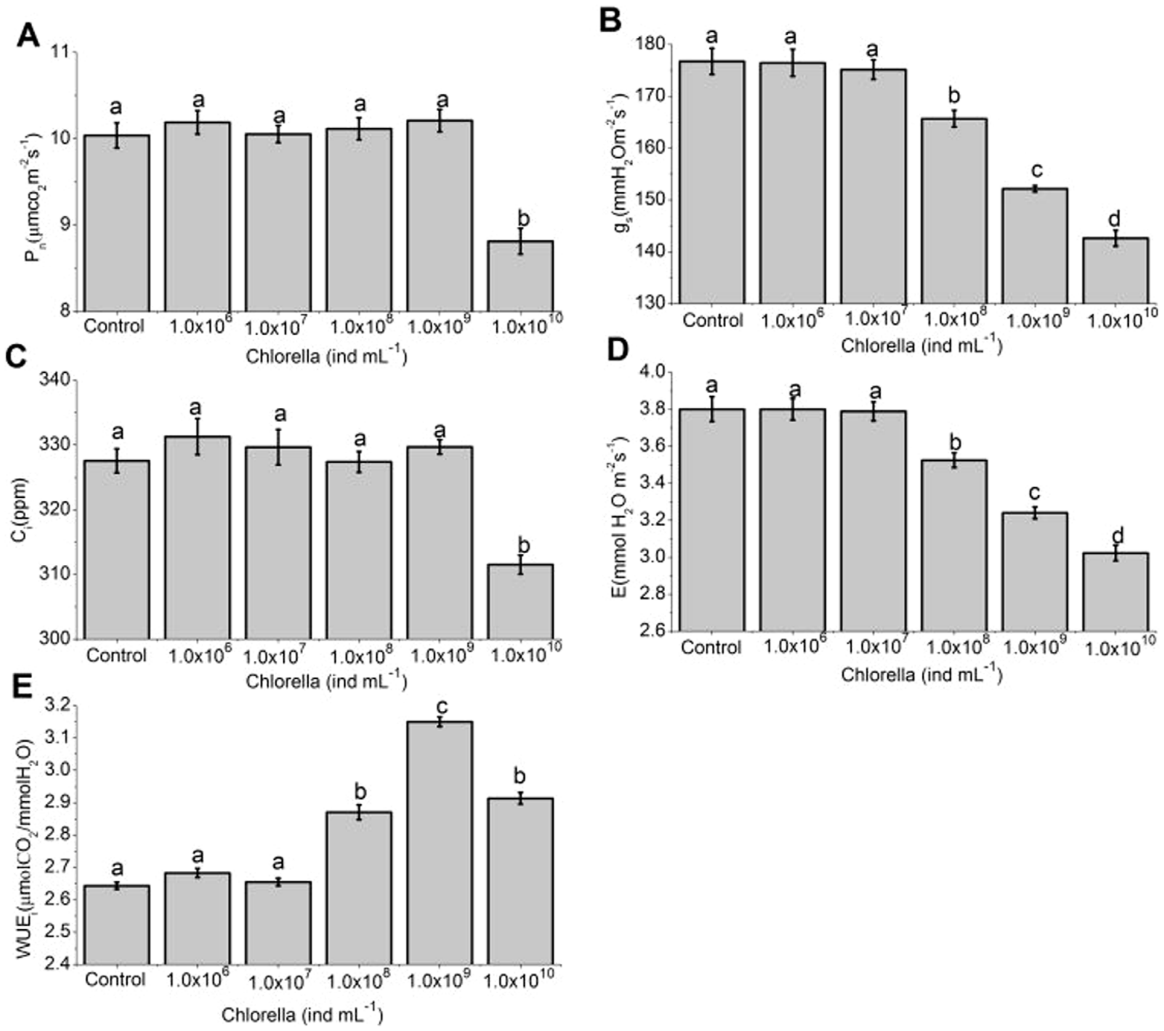
The effects of Chlorella on gas exchange in leaves of broad bean. Alterations in (A) net photosynthetic rate (P_n_), (B) stomatal conductance (g_s_), (C) intercellular CO_2_ concentration (C_i_), (D) transpiration rate (E), and (E) instantaneous intrinsic water use efficiency (WUE_i_), of broad bean leaves at 48 h after treatment with different concentrations of Chlorella suspension (1.0×10^6^, 1.0×10^7^, 1.0×10^8^, 1.0×10^9^ and 1.0×10^10^ ind mL^−1^). These data are the mean ± SE (n = 14 per bar). Different letters above the bars indicate mean values that are significantly different from one another as determined by ANOVA (LSD test, *P*<0.05).

To survey the effects of Chlorella on plant photosynthetic gas-exchange, 1.0×10^6^, 1.0×10^7^, 1.0×10^8^, 1.0×10^9^ and 1.0×10^10^ ind mL^−1^ of Chlorella suspension were evenly sprayed onto leaves of broad beans. After 48 h, the stomatal apertures were reduced by 0.4% (*P* = 0.999), 1.1% (*P* = 0.863), 15.2% (*P*<0.001), 21.8% (*P*<0.001), and 27.2% (*P*<0.001), respectively (Figure 4), the g_s_ was severally reduced by 1.6‰ (*P* = 0.919), 8.9‰ (*P* = 0.563), 6.3% (*P*<0.001), 13.9% (*P*<0.001) and 19.3% (*P*<0.001), and the E was reduced by 0.2‰ (*P* = 0.992), 3‰ (*P* = 0.872), 7.3% (*P*<0.001), 14.7% (*P*<0.001) and 20.4% (*P*<0.001), respectively (Figure 5). Furthermore, the P_n_ and C_i_ were hardly affected when the concentrations of Chlorella suspension were less than or equal to 1.0×10^9^ ind mL^−1^ (*P*>0.05), while were significantly inhibited at a higher concentration (*P*<0.001) (Figure 5). As a result, the WUE_i_ were separately improved by 1.5% (*P* = 0.082), 4‰ (*P* = 0.600), 8.6% (*P*<0.001), 19.2% (*P*<0.001) and 10.2% (*P*<0.001) (Figure 5).

### Chlorophyll fluorescence and content analysis

To investigate whether plant photosynthetic activities are influenced by Chlorella, leaf chlorophyll fluorescence was tested on the same leaves used for gas exchange analysis. No statistical alterations in the values of minimal fluorescence (F_0_) and maximal fluorescence (F_m_) were observed after different treatments (Table S1). As a result, there was no pronounced change in maximal photochemical efficiency, F_v_/F_m_ (*P*>0.05) (Table 1). Furthermore, other chlorophyll fluorescence parameters such as effective quantum yield of PSII (ΦPSII), non-photochemical quenching (NPQ), the coefficient for photochemical quenching (qP) and electron transportation rate (ETR) were not significantly affected by different concentrations of Chlorella suspension (*P*>0.05) (Table 1). Moreover, as noted in Table 1, the contents of chlorophyll a, chlorophyll b and total chlorophyll and the ratio of chlorophyll a/b were not different among treatments (*P*>0.05).

**Table 1:** Photosynthetic activity and chlorophyll content of broad bean leaves after treatment with different concentrations of Chlorella suspension raging from 0 to 1.0×10^10^ ind mL^−1^ for 48 h.

Parameters	Control	1.0×10^6^	1.0×10^7^	1.0×10^8^	1.0×10^9^	1.0×10^10^
**F_v_/F_m_**	0.79±0.004^a^	0.78±0.003^a^	0.79±0.004^a^	0.79±0.002^a^	0.79±0.002^a^	0.79±0.002^a^
**ΦPSII**	0.63±0.004^a^	0.63±0.002^a^	0.62±0.005^a^	0.63±0.003^a^	0.62±0.002^a^	0.62±0.002^a^
**NPQ**	0.78±0.020^a^	0.78±0.014^a^	0.80±0.009^a^	0.78±0.007^a^	0.78±0.005^a^	0.79±0.007^a^
**qP**	0.92 ±0.003^a^	0.91±0.001^a^	0.91±0.002^a^	0.91±0.003^a^	0.92±0.001^a^	0.91±0.005^a^
**ETR**	44.14±0.396^a^	43.77±0.304^a^	43.37±0.486^a^	43.12±0.321^a^	43.92±0.244^a^	43.96±0.402^a^
**Chl. a**	1.84±0.032^a^	1.84±0.015^a^	1.89±0.023^a^	1.87±0.022^a^	1.84±0.009^a^	1.87±0.016^a^
**Chl. b**	0.79±0.020^a^	0.75±0.018^a^	0.78±0.018^a^	0.79±0.020^a^	0.75±0.019^a^	0.76±0.027^a^
**Chl. a+b**	2.63±0.049^a^	2.60±0.025^a^	2.67±0.037^a^	2.66±0.036^a^	2.59±0.021^a^	2.64±0.038^a^
**Chl. a/b**	2.36±0.039^a^	2.46±0.059^a^	2.43±0.043^a^	2.38±0.055^a^	2.49±0.062^a^	2.48±0.082^a^

F_v_/F_m_, maximum quantum yield of photosystem II (PSII); ΦPSII, effective quantum yield of PSII; NPQ, non-photochemical quenching; qP, photochemical quenching; ETR, electron transportation rate; Chl. a, Chl. b, Chl. a + b, chlorophyll a, chlorophyll b, total chlorophyll content, expressed as mg per g fresh weight; Chl. a/b, ratio of chlorophyll a to b. Each value represents the mean ± SE (n = 14). Means estimates with same letters are not significantly different among treatments as determined by ANOVA (LSD test, *P*<0.05).

## Discussion

### The effects of Chlorella on stomatal movement in broad bean

Stomatal openings have long been recognized as passive access to inner leaf tissues for many plant bacteria. However, recent studies have revealed that potential microbes can induce stomatal closure by activating a series of signaling transduction pathways [8], [9], [12], [14], [15], [24]. In the present study, we found that Chlorella as an available autotrophic microorganism induced stomatal closure in epidermal strips of broad beans in a dose-and time-dependent manner, arriving at the maximum effect at 1.0×10^9^ ind mL^−1^ of Chlorella suspension and 2 h after treatment (Figure 1). These results further validate that microbes can actively trigger stomatal closure. Mounting evidence suggests that microbe-triggered stomatal closure relies on the perception of MAMPs by pattern recognition receptors (PRRs) [54]–[56]. MAMPs are conserved molecules on the surface of microorganisms, such as flg22, elf18, LPS, fungal cell wall derived chitin, ergosterol, glucans and chitosan oligosaccharides, yeast elicitor, plant cell wall derived oligogalacturonic acid, as well as various glycopeptides and glycoproteins, most of which are capable of inducing stomatal closure [57]–[59]. It is known that Chlorella cell wall and extracellular organic matter (EOM) attached at the Chlorella cell surface are mainly composed of polysaccharides, glycoproteins, lipids, glucosamine, chitin-like glycans and other matrix [60], [61]. Consequently, we speculate that some components of Chlorella cell wall and EOM are involved in Chlorella-triggered stomatal closure. Further work will be done to unveil which component induces stomatal closure.

### The effects of Chlorella on ROS production in broad bean

In plant cells, ROS functions as an integral intermediate in MAMPs- and ABA-dependent stomatal closure [9], [24], [32], [34], [42]. Our work primarily indicated that Chlorella-induced stomatal closure was accompanied by ROS production (Figure 3), which is similar to live pathogenic microbes, yeast and MAMPs-induced stomatal closure [8], [9], [12], [15], [24]. ROS production in guard cells is mediated by various enzymes in response to different stimuli, including NADPH oxidase, peroxidase, xanthine oxidase, oxalate oxidase and amine oxidase [25]–[28], [52], [62], [63]. To clarify which enzyme catalyzes ROS production mediating Chlorella-induced stomatal closure, we further assessed the effects of Chlorella on stomatal movement and ROS production with pharmacological inhibitors or scavenger such as DPI, SHAM and CAT [24], [42]. We observed that Chlorella-induced stomatal closure was almost completely reversed by an H_2_O_2_ scavenger, CAT, strongly inhibited by an NADPH oxidase inhibitor, DPI, while slightly suppressed by a peroxidase inhibitor, SHAM (Figure 2). In addition, it was shown that the Chlorella-induced ROS production was completely abolished by CAT, largely restrained by DPI, whereas hardly affected by SHAM (Figure 3). Taken together, these results suggest that Chlorella-induced stomatal closure is mediated by ROS production mainly via DPI-sensitive plasma membrane NADPH oxidase but not SHAM-sensitive peroxidase. It is analogous to ABA-, MeJA-, ozone-, darkness-, ethylene-, AITC-, low dose of UV-B-, flg22-, LPS- and elf18-induced stomatal closure modulated by DPI-sensitive NADPH oxidase [9], [29]–[36], while different from SA-, high dose of UV-B-, chitosan-, YEL-, methylglyoxal- and yeast-triggered stomatal closure via SHAM-sensitive peroxidase [15], [24], [38]–[41]. These results will provide insights for the prediction of elicitors from Chlorella. In plants, the NADPH oxidase respiratory burst oxidase homologs (RBOHs) are encoded by 10 different RBOHs genes, four of which have been characterized, namely *RBOHB*, *RBOHC*, *RBOHD* and *RBOHF* [64]. Among four RBOHs, RBOHD is the primary NADPH oxidase responsible for the rapid ROS production in response to MAMPs, RBOHF on the other hand, is the main ROS-producing enzymes mediating ABA-dependent ROS production [65]. Although our work showed that Chlorella-induced ROS production was mainly dependent on NADPH oxidase, which RBOH catalyzes the generation of ROS remains unsloved. Mechanical studies have revealed that stomatal closure triggered by biotic and abiotic stresses is related to the ABA-mediated complex network of signalling events, including activation of G-proteins and the guard cell specific OST1 kinase, generation of ROS and nitric oxide (NO), elevation of cytosolic Ca^2+^ levels and Ca^2+^ oscillations, protein phosphorylation/dephosphorylation, reorganization of the cytoskeleton, elevation of cytosolic pH, and activation of cation and anion channels [66]. Besides ROS, whether other components of ABA signaling pathways are involved in Chlorella-induced stomatal closure need to be explored in the future. The stomatal system of *Arabidopsis* mutant will provide an attractive tool for dissecting novel aspects of signaling events.

### Involvement of Chlorella-induced stomatal closure in the improvement of WUE_i_ in broad bean

Stomata play a prominent role in controlling gas exchanges between plants and environments. Numerous studies have demonstrated that moderate stomatal closure is an extraordinary adaptation to various environmental stresses in plants [7], [67]–[70]. When the plant encounters mild stress, the leaf stomatal conductance usually decreases earlier than photosynthesis, leading to the increase of WUE_i_ in plants [3], [4], [67], [71]. Therefore, Chlorella-induced stomatal closure can be expected to improve WUE_i_ of plants by preventing more water loss through transpiration. Our studies showed that Chlorella caused reductions in leaf stomatal aperture, g_s_ and E until the concentration reached 1.0×10^8^ ind mL^−1^ in intact leaves (Figure 4 and Figure 5). The images in Figure 4 provided further visible proof that reductions in transpiration rate and stomatal conductance in whole leaves when the concentration reached 1.0×10^8^–1.0×10^10^ ind mL^−1^ were only due to the Chlorella-induced stomatal closure rather than mechanical obstruction of stomata caused by stacked Chlorella cells. Comparing the results in Figure 1, Figure 4 and Figure 5, it could been seen that there was a linear response in stomatal closure when floating epidermal peels on increasing Chlorella concentration from 1.0×10^6^ up to 1.0×10^9^ ind mL^−1^ (Figure 1), however, stomatal aperture, transpiration rate and stomatal conductance (Figure 4 and Figure 5) remained unchanged up to 1.0×10^7^ ind mL^−1^ in intact leaves. These differences between stomata in intact leaves and epidermal peels responding to various treatments may be attributed to the stomatal sensitivity to different stimuli because of the structure discrepancies. It agreed with a previous study that stomata in epidermal peels of *Commelina communis* L. were more sensitive to ABA than that in intact leaves [72]. The study revealed that the cuticle and mesophyll of the whole leaf might limit the stomatal sensitivity to applied stimulus [72]. Furthermore, the P_n_ and C_i_ were hardly affected after treatment with less than or equal to 1.0×10^9^ ind mL^−1^ of Chlorella suspension, whereas were significantly repressed at a higher concentration (Figure 5). As a consequence, the WUE_i_ of broad beans was improved greatly when proper concentrations of Chlorella suspension were applied, while less affected at higher concentrations due to the inhibition of leaf P_n_ (Figure 5). Previous studies have indicated that photosynthesis is not only restricted by stomatal limitations of the CO_2_ influx but also by non-stomatal limitations that influence activities of photosynthetic reactions center such as photosynthetic apparatus, ATP synthesis, and electron transfer [73]–[76]. The comprehensive information about the photosynthetic activity can be obtained from chlorophyll fluorescence and content analysis [73]. Our data demonstrated that no pronounced alterations in the chlorophyll content and fluorescence parameters, including F_v_/F_m_, ΦPSII, NPQ, qP, and ETR, were detected 48 h after application of various concentrations of Chlorella suspension (Table 1). These phenomena suggest that short-term Chlorella treatment neither affect the photosynthetic transport of electrons nor lead to photoinhibition at the PSII complexes [44], [75]. The data together with the changes of P_n_ and C_i_ reveal that inhibition of photosynthesis at a higher concentration of Chlorella suspension is only attributed to a decrease in CO_2_ transfer from atmosphere. To sum up, these results initially present that optimal concentrations of Chlorella suspension improve the WUE_i_ in plants after exogenous application, mainly ascribing to a reduction in stomatal conductance above the threshold, under which transpiration rates decrease greatly whereas photosynthetic rates are barely affected.

In conclusion, Chlorella could be sensed by guard cell to trigger partial stomatal closure via NADPH oxidase-dependent ROS production and improve WUE_i_ in *V. faba* when applied as foliar spray. In addition, heated-killed Chlorella cells could trigger stomatal closure and improve WUE_i_, just as live Chlorella cells did (Figure S2 and Figure S3). Further work will be needed to comprehensively understand the effects of Chlorella on stomatal movement and WUE_i_ of a wide of agricultural crops. It will provide scientific evidence for the development of new bio-antitranspirant and advancement of water-saving technology for solving fresh water scarcity.

## Supporting Information

Figure S1 **Effects of 20 µM DPI, 2 mM SHAM and 100 UmL^−1^ CAT on stomatal aperture in** ***V. faba*** **.** Epidermal peels of broad beans preincubated for 2 h in opening buffer under light were treated with 20 µM DPI, 2 mM SHAM and 100 UmL^−1^ CAT and stomatal apertures were measured after 2 h. These data are the mean ± SE of the representative results from three biological repeats (n = 150 per bar). Same letters above the bars indicate mean values that are not significantly different from one another as determined by ANOVA (LSD test, *P*<0.05).
(TIF)

Figure S2 **Heated-killed Chlorella-triggered stomatal closure in** ***V. faba*** **.** (A) The dosage effect of heated-killed Chlorella-induced stomatal closure in epidermal peel experiments. Epidermal peels of broad beans preincubated for 2 h in opening buffer under light were treated with different concentrations of heated-killed Chlorella suspension, and stomatal apertures were measured after 2 h. These data are the mean ± SE of three biological replicates (n = 150 per bar). (B) The dosage effect of heated-killed Chlorella-triggered stomatal closure in intact leaves. After evenly spraying various concentrations of heated-killed Chlorella suspension and water onto the leaves of broad bean for 48 h, the epidermal strips were peeled off and immediately observed under a microscope. Each bar represents the mean ± SE of the representative results (n = 120). Different letters above the bars indicate mean values that are significantly different from one another as determined by ANOVA (LSD test, *P*<0.05).
(TIF)

Figure S3 **The effects of Heated-killed Chlorella on instantaneous water use efficiency (WUE_i_) in** ***V. faba*** **.** The changes in instantaneous intrinsic water use efficiency (WUE_i_) of broad bean leaves 48 h after treatment with different concentrations of heated-killed Chlorella suspension (1.0×10^6^, 1.0×10^7^, 1.0×10^8^, 1.0×10^9^ and 1.0×10^10^ ind mL^−1^). These data are the mean ± SE (n = 14 per bar). Different letters above the bars demonstrate mean values that are significantly different from one another as determined by ANOVA (LSD test, *P*<0.05).
(TIF)

Table S1 **The minimal fluorescence (F_0_) and maximal fluorescence (F_m_) of broad bean leaves after treatment with different concentrations of Chlorella suspension raging from 0 to 1.0×10^10^ ind mL^−1^ for 48 h.** Each value represents the mean ± SE (n = 14). Means estimates with same letters are not significantly different among treatments as determined by ANOVA (LSD test, *P*<0.05).
(DOC)
