# Supplementary figures and images for: Chlorella Induces Stomatal Closure via NADPH Oxidase-Dependent ROS Production and Its Effects on Instantaneous Water Use Efficiency in Vicia faba
Source: PLoS One. 2014 Mar 31;9(3):e93290. doi: 10.1371/journal.pone.0093290 (PMC3970962; doi:10.1371/journal.pone.0093290)

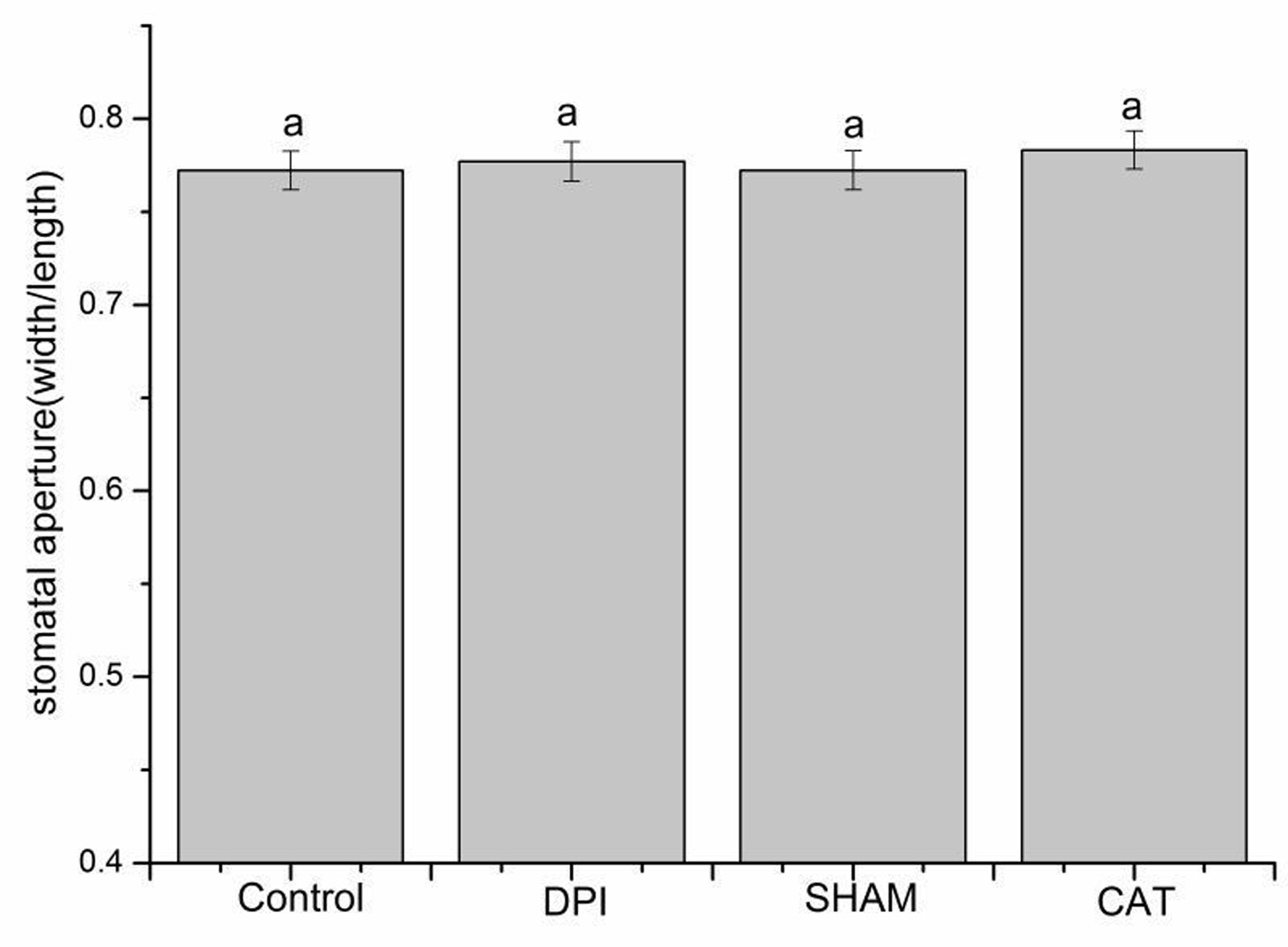

Supplement: Figure S1 — Effects of 20 µM DPI, 2 mM SHAM and 100 UmL−1 CAT on stomatal aperture in V. faba . Epidermal peels of broad beans preincubated for 2 h in opening buffer under light were treated with 20 µM DPI, 2 mM SHAM and 100 UmL−1 CAT and stomatal apertures were measured after 2 h. These data are the mean ± SE of the representative results from three biological repeats (n = 150 per bar). Same letters above the bars indicate mean values that are not significantly different from one another as determined by ANOVA (LSD test, P<0.05). (TIF) [file pone.0093290.s001.tif]

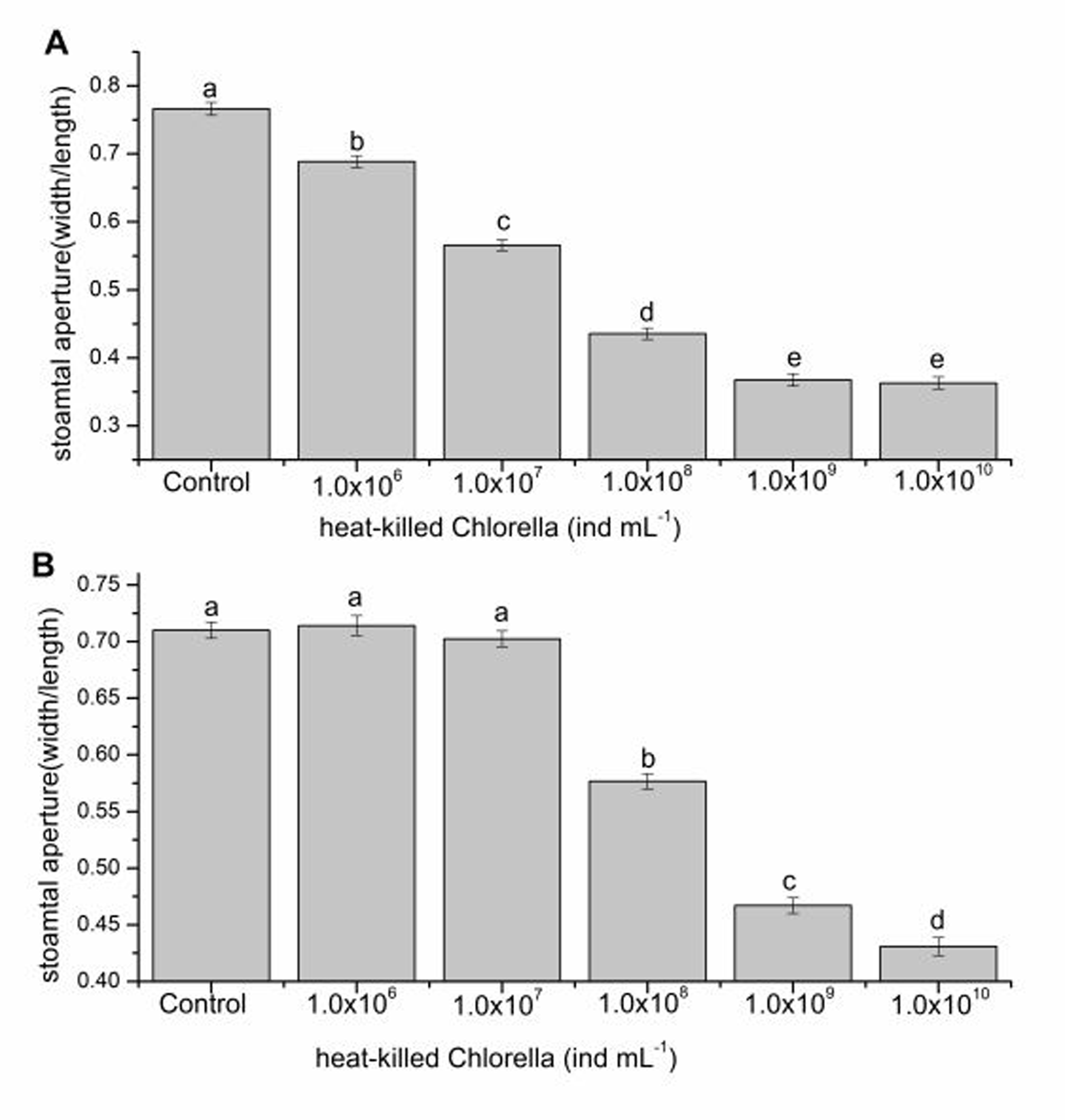

Supplement: Figure S2 — Heated-killed Chlorella-triggered stomatal closure in V. faba . (A) The dosage effect of heated-killed Chlorella-induced stomatal closure in epidermal peel experiments. Epidermal peels of broad beans preincubated for 2 h in opening buffer under light were treated with different concentrations of heated-killed Chlorella suspension, and stomatal apertures were measured after 2 h. These data are the mean ± SE of three biological replicates (n = 150 per bar). (B) The dosage effect of heated-killed Chlorella-triggered stomatal closure in intact leaves. After evenly spraying various concentrations of heated-killed Chlorella suspension and water onto the leaves of broad bean for 48 h, the epidermal strips were peeled off and immediately observed under a microscope. Each bar represents the mean ± SE of the representative results (n = 120). Different letters above the bars indicate mean values that are significantly different from one another as determined by ANOVA (LSD test, P<0.05). (TIF) [file pone.0093290.s002.tif]

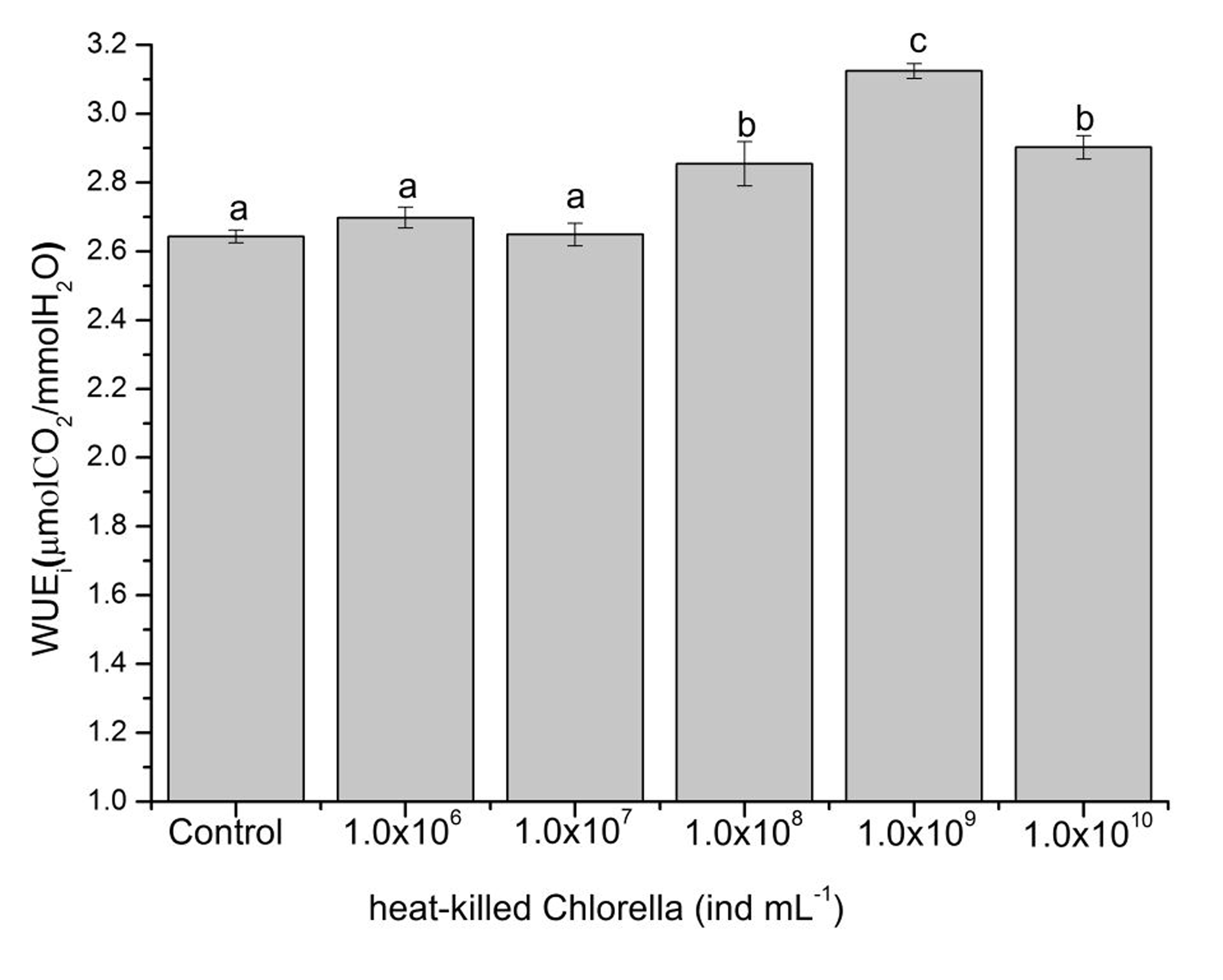

Supplement: Figure S3 — The effects of Heated-killed Chlorella on instantaneous water use efficiency (WUEi) in V. faba . The changes in instantaneous intrinsic water use efficiency (WUEi) of broad bean leaves 48 h after treatment with different concentrations of heated-killed Chlorella suspension (1.0×10^6^, 1.0×10^7^, 1.0×10^8^, 1.0×10^9^ and 1.0×10^10^ ind mL−1). These data are the mean ± SE (n = 14 per bar). Different letters above the bars demonstrate mean values that are significantly different from one another as determined by ANOVA (LSD test, P<0.05). (TIF) [file pone.0093290.s003.tif]
