# Supplementary material for: Chlorella Induces Stomatal Closure via NADPH Oxidase-Dependent ROS Production and Its Effects on Instantaneous Water Use Efficiency in Vicia faba
Source: PLoS One. 2014 Mar 31;9(3):e93290. doi: 10.1371/journal.pone.0093290 (PMC3970962; doi:10.1371/journal.pone.0093290)
Supplement: Table S1 — The minimal fluorescence (F0) and maximal fluorescence (Fm) of broad bean leaves after treatment with different concentrations of Chlorella suspension raging from 0 to 1.0×10^10^ ind mL−1 for 48 h. Each value represents the mean ± SE (n = 14). Means estimates with same letters are not significantly different among treatments as determined by ANOVA (LSD test, P<0.05). (DOC) [file pone.0093290.s004.doc]

**Table S1. The minimal fluorescence (F_0_) and maximal fluorescence (F_m_) of broad bean leaves after treatment with different concentrations of Chlorella suspension raging from 0 to 1.0×10^10^ ind mL^-1^ for 48 h.**

| **Parameters** | **Control** | **1.0×10^6^** | **1.0×10^7^** | **1.0×10^8^** | **1.0×10^9^** | **1.0×10^10^** |
| --- | --- | --- | --- | --- | --- | --- |
| **F_0_** | 0.104±0.0008^a^ | 0.104±0.0007^a^ | 0.104±0.007^a^ | 0.104±0.007^a^ | 0.104±0.007^a^ | 0.104±0.008^a^ |
| **F_m_** | 0.489±0.0320^a^ | 0.482±0.0263^a^ | 0.496±0.0296^a^ | 0.495±0.0209^a^ | 0.493±0.0183^a^ | 0.489±0.0129^a^ |

Each value represents the mean ± SE (n=14). Means estimates with same letters are not significantly different among treatments as determined by ANOVA (LSD test, *P*<0.05).
